# Letter to the editor: Wide indication for SARS-CoV-2-testing allowed identification of international risk areas during the early phase of the COVID-19 pandemic in Germany

**DOI:** 10.2807/1560-7917.ES.2020.25.23.2001119

**Published:** 2020-06-11

**Authors:** Ute Rexroth, Osamah Hamouda, Johanna Hanefeld, Bettina Ruehe, Lothar H Wieler, Lars Schaade

**Affiliations:** 1 Robert Koch Institute, Berlin, Germany

**Keywords:** COVID-19, SARS-CoV-2, pandemic, testing strategy, international risk areas

**To the editor:** In their recent rapid communication in ‘Rapid response infrastructure for pandemic preparedness in a tertiary care hospital: lessons learned from the COVID-19 outbreak in Cologne, Germany, February to March 2020’ [1], Augustin et al. describe how a rapid response infrastructure was established to manage large numbers of suspected and confirmed coronavirus disease (COVID-19) cases in a German university hospital.

Their description is an example of the exceptional effort and adaptability of healthcare providers, that helped limit nosocomial transmission and hospital outbreaks in Germany in the first months of the COVID-19 pandemic. It also demonstrates that timely case-based information provided by clinicians and laboratories is invaluable for the development and adaptation of data-driven guidelines. Such information is therefore collected via the German national surveillance system. The Robert Koch Institute (RKI), Germany’s national public health institute, used the information on the likely place of infection as a criterion for the definition of international risk areas (alongside local incidence, epidemic trend, measures taken, origin of internationally exported cases and transport connectivity to Germany).

We would thus like to point out, that Augustin et al. did not act against, but in compliance with national guidelines when they tested patients without travel history to a declared risk area for severe acute respiratory syndrome coronavirus 2 (SARS-CoV-2). On the contrary, RKI relies on clinicians to remain vigilant, to include COVID-19 in their differential diagnosis and to routinely question patients about their travel history.

On 17 January 2020, RKI first published a guidance for doctors on measures and testing criteria for suspected COVID-19 cases [2]. Therapeutic freedom, including diagnostic decision-making of medical practitioners is guaranteed by German law. Similar to all technical guidelines by RKI, the guidance on testing criteria and measures is not binding. It aims to provide orientation for clinicians as to when testing for SARS-CoV-2 is especially recommended, and when suspected COVID-19-cases require specific infection prevention and control measures as well as immediate notification to local public health authorities. This document is being constantly adapted to the developing epidemiological situation. The version history is available online at: https://edoc.rki.de/handle/176904/6459 and https://edoc.rki.de/handle/176904/6484.11.

Until 11 March 2020, COVID-19 was not considered a pandemic by the World Health Organization (WHO). It is important to note that towards the end of February and beginning of March, the influenza season was still active and full SARS CoV-2 testing capacity was not yet established in Germany. It would thus not have been feasible to recommend SARS-CoV-2 testing for everybody irrespective of clinical symptoms and risk exposure.

Risk exposure was defined as (i) having had contact to a confirmed case or (ii) travel history to a risk area. From 12 February, SARS-CoV-2-testing was additionally recommended for individuals with clinical signs (without alternative explanation), who did not report risk exposure as defined above, but rather had a travel history to any region in the world where COVID-19-cases had been confirmed, including specific regions in Germany. To inform both health professionals and the public, RKI published updated case numbers worldwide including considerations on which geographical regions were actually considered ‘risk areas’.

At the end of February 2020, RKI re-emphasised the importance of including COVID-19 in the differential diagnosis in individuals, who had a travel history to a region where COVID-19-cases had been confirmed, even if it was not defined as ‘risk area’. However, in contrast to individuals with travel history to ‘risk areas’, these patients were not regarded as suspected cases. Hence, it was not recommended to strictly isolate them and to immediately notify them to public health authorities.

The valuable description of Augustin et al. demonstrates that RKI’s national guidelines were known and applied in German hospitals to guide clinicians’ decision-making and management of potential cases. The paper also demonstrates the value of exceptional commitment by clinicians. Moreover, it suggests that during the evolving pandemic there may have been delays in frequent updates reaching frontline clinicians. Augustin et al.’s case study highlights the importance of well-established communication channels between public health authorities and healthcare providers, and the need for their further improvement to even better prepare for the next wave of COVID-19.
